# Discriminating between ADHD adults and controls using independent ERP components and a support vector machine: a validation study

**DOI:** 10.1186/1753-4631-5-5

**Published:** 2011-07-19

**Authors:** Andreas Mueller, Gian Candrian, Venke Arntsberg Grane, Juri D Kropotov, Valery A Ponomarev, Gian-Marco Baschera

**Affiliations:** 1 Brain and Trauma Foundation Grisons, Poststrasse 22, 7000 Chur, Switzerland; 2 Neuropsychological Service, Helgeland Hospital, Mosjøen, Norway; 3 Institute of the Human Brain of Russian Academy of Sciences, St. Petersburg, Russian Federation; 4 Department of Computer Science, ETH Zurich, Switzerland

## Abstract

**Background:**

There are numerous event-related potential (ERP) studies in relation to attention-deficit hyperactivity disorder (ADHD), and a substantial number of ERP correlates of the disorder have been identified. However, most of the studies are limited to group differences in children. Independent component analysis (ICA) separates a set of mixed event-related potentials into a corresponding set of statistically independent source signals, which are likely to represent different functional processes. Using a support vector machine (SVM), a classification method originating from machine learning, this study aimed at investigating the use of such independent ERP components in differentiating adult ADHD patients from non-clinical controls by selecting a most informative feature set. A second aim was to validate the predictive power of the SVM classifier by means of an independent ADHD sample recruited at a different laboratory.

**Methods:**

Two groups of age-matched adults (75 ADHD, 75 controls) performed a visual two stimulus go/no-go task. ERP responses were decomposed into independent components, and a selected set of independent ERP component features was used for SVM classification.

**Results:**

Using a 10-fold cross-validation approach, classification accuracy was 91%. Predictive power of the SVM classifier was verified on the basis of the independent ADHD sample (17 ADHD patients), resulting in a classification accuracy of 94%. The latency and amplitude measures which in combination differentiated best between ADHD patients and non-clinical subjects primarily originated from independent components associated with inhibitory and other executive operations.

**Conclusions:**

This study shows that ERPs can substantially contribute to the diagnosis of ADHD when combined with up-to-date methods.

## Background

Attention-deficit hyperactivity disorder (ADHD) is a clinically heterogeneous disorder that is associated with high financial costs, stress to families and interpersonal relationships, and adverse academic and vocational outcomes [1]. According to the Diagnostic and Statistical Manual of Mental Disorders (DSM-IV; [2]), attention-deficit hyperactivity disorder (ADHD) is characterized by varying levels of inattention, hyperactivity, and impulsivity symptoms. ADHD was considered a childhood disorder which does not progress into adulthood [3]. By now, it is known that more than a half of all ADHD children continue to display clinically significant symptoms after reaching adulthood [4,5]. Nevertheless, the presenting symptoms change over time and adults with ADHD are more likely to complain of difficulties involving executive functions, rather than of hyperactivity [6].

One of the most influential theoretical models of ADHD postulates that a deficit in behavioral inhibition is the core of ADHD [7,8]. According to this theory, behavioral inhibition is considered to be the foundation of the executive functions, which in turn influence the motor system. Kropotov [9] distinguishes four types of executive operations: engagement operations, disengagement operations, working memory, and monitoring operations. These operations perform on representations of actions by initiating and suppressing actions, by storing plans of actions and by comparing ongoing actions and performance outcomes with internal goals and standards [10]. It is assumed that these operations are subserved by different neuronal mechanisms and - as well as sensory functions - are reflected in different components of scalp-recorded evoked potentials.

In contrast to spontaneous EEG activity, event-related potentials (ERPs) reflect phasic activity of cortical neurons. They are electrophysiological responses to an internal or external stimulus and are obtained by averaging the brain's electrical response to the stimuli over a number of trials. ERPs exhibit a number of characteristic peaks and troughs - their components - which are associated with underlying stages of sensory-related and action-related information flow in various cortical areas. A particular ERP component can be characterized by its eliciting condition, polarity, latency and scalp distribution (topography). There are a multitude of tasks that are used to elicit ERPs. These tasks cover a variety of distinct cognitive operations such as the detection and recognition of stimuli, updating working memory and the initiation, suppression and monitoring of action. In the context of ADHD, ERPs have been investigated in a large number of studies and a substantial number of ERP correlates of ADHD could be identified [11]. Being primarily designed for the study of neurophysiological mechanisms of the executive functions of the brain [12], the go/no-go task represents a frequently used paradigm for the study of brain functioning in patients with ADHD. Subjects are instructed to perform an action in response to one type of stimuli (go) and to suppress the action in response to another type of stimuli (no-go).

Applying this paradigm, several group differences relating to different ERP components have been found between ADHD and control subjects. Smith et al. [13], when examining auditory ERPs, detected topographic differences in components associated with early stimulus processing (P1, N1, P2) as well as in the inhibition-related N2 component. Furthermore, ADHD children showed earlier P2 and N2 peaks compared to control children. The results were interpreted in terms of ADHD children showing problems with sensory registration and identification of stimuli. Further, it was suggested that ADHD children have to trigger the inhibition process earlier and more strongly in order to achieve the same behavioral performance as the controls. Using an auditory go/no-go task as well, Broyd et al. [14] found enhanced N1 and P2 amplitudes and reduced N2 amplitudes in children with ADHD when compared to controls. The results were interpreted in terms that ADHD patients exhibit inhibitory deficiencies. Increased P2 and reduced N2 amplitudes in children with ADHD were also found by Johnstone and Clarke [15] when using a visual go/no-go task. Furthermore, the ADHD group showed a more anterior P3 to nogo stimuli, relative to go stimuli, when compared to controls. ERP studies on adults with ADHD are scarce. Prox et al. [16], using a visual go/no-go task, found increased N1 and N2 amplitudes in their adult ADHD group. They interpreted these results in terms of the patients compensating for their impairments by shifting more attention to the task and by intensifying the inhibition of their responses in order to be successful in accomplishing the task. Fallgatter et al. [17] reported a reduced nogo anteriorisation, a neurophysiological correlate of prefrontal response control that has been suggested to reflect activation of the anterior cingulate cortex [18], and a reduced increase of fronto-central P3 amplitudes in nogo trials compared to controls. Along with the results of their ERP source localizations they concluded that patients with ADHD-related psychopathology are characterized by a prefrontal brain dysfunction related to response inhibition and/or cognitive control.

Investigating the functional significance of ERP components is the focus of ongoing research. However, given that ERP components are highly specific to the behavioural paradigm used for their elicitation, associating ERP components with a clear functional meaning poses a serious challenge. For instance, Folstein and Van Petten [19] concluded that, even when limited to visual studies, the frontocentral N2 component of the ERP has multiple functional correlates. One way to approximate the functional meaning is to localize the ERP component's current source by means of EEG source localization techniques such as sLORETA [20]. A further method of approaching the functional meaning of specific ERP components is provided by independent component analysis (ICA). By means of ICA, a set of mixed potentials measured at the scalp is separated into a corresponding set of statistically independent source signals [21,22]. Each of these sources, referred to as independent components, is characterized by a fixed scalp topography and an independent time course. Using ICA, Kropotov and Ponomarev [23] were able to decompose the N2 ERP wave into three different independent components associated with distinct functional operations.

There are a limited number of studies investigating whether ERP measures of brain function are of diagnostic utility and can be used to reliably classify normal controls versus patients with ADHD (for a review, see [11]). The classification accuracy of these investigations is moderate, achieving 71 to 81% correct identifications. In a more recent attempt to separate these diagnostic groups, Smith et al. [24] employed an active auditory oddball paradigm and combined topographic ERP component amplitude and latency data with behavioral performance measures. Applying discriminant function analysis, control children aged 8-12 years could be distinguished from children with ADHD of the same age with 73.3% overall classification accuracy. Classification accuracy for the adolescent groups aged 13-18 years was only slightly above chance level.

In recent years, new non-linear methods such as support vector machines (SVM), a group of supervised learning methods that can be applied to classification, have emerged in the field of electroencephalography [25-27]. Using a set of pre-classified training samples, each belonging to one of two classes, a SVM algorithm builds a model that predicts whether new test samples fall into one class or into the other. In the present case, the SVM analyzes the ERP features of a number of subjects, each known to belong to either the ADHD or control group, and predicts the membership of further subjects to one of these groups on the basis of an optimal feature set. Classification is performed by constructing a hyperplane that maximizes the separating margin between the closest samples of the two classes. The samples can be separated by non-linear curves by transforming the data into a higher dimensional space. When applying such non-linear classifiers, nonlinear relationships in the feature data that are not obvious may be found. Merzagora et al. [28] investigated the relative performances of different linear and non-linear classifiers with regard to their ability to accurately classify ERP responses to target and to non-target stimuli. Based on different selections of P3 and N2 features, non-linear and non-parametric classifiers (quadratic classifier, multi-layer perceptron neural network, support vector machine) outperformed linear classifiers (Euclidean classifier, Mahalanobis discriminant, Fisher's linear discriminant), reaching an accuracy of more than 90%. Apart from the superior performance of non-linear classifiers, the authors concluded that the automatic characterization of target ERPs can provide an objective approach for detecting and diagnosing abnormalities in clinical populations.

In a recent study, Mueller et al. [29] were able to accurately classify ADHD patients and controls on the basis of independent ERP components. Using a non-linear SVM classifier, classification accuracy was 92%. In contrast to the present study, the independent component's activation curves were constructed using generic spatial filters which were built on the basis of ERPs derived from a large set of healthy subjects. The robustness of these filters notwithstanding, this approach may induce spurious latency or amplitude effects in the time courses of the independent components. In the present study, ICA was performed on a collection of individual ERP responses from both an ADHD and a control group, and individual independent component activation curve features were used in order to discriminate between the two groups by means of a non-linear support vector machine classifier. Furthermore, classification performance was validated using an independently tested ADHD sample.

The purpose of this study was threefold. The study was aimed at examining the utility of independent ERP components in classifying adult ADHD patients and non-clinical subjects, at separating a combination of features which prove most informative for the classification and at validating the predictive power of the SVM classifier by means of an independent ADHD sample. In more general terms, this study is part of a work aiming at identifying biological markers in terms of personalized medicine.

## Methods

### Subjects

Two age- and sex-matched groups participated in the study, each consisting of 37 female and 38 male subjects aged between 20 and 50. The mean age in the ADHD group was 36.05 years (SD 8.42). ADHD subjects were recruited by advertising the study in the media and by notifying psychiatrists and ADHD associations of the study. Inclusion in the ADHD group was based on the DSM-IV criteria for ADHD [2], assessed in a diagnostic interview [30]. 24 subjects met the DSM-IV criteria for the ADHD combined type, 42 subjects met the criteria for the ADHD predominantly inattentive type, and 9 subjects met the criteria for the ADHD predominantly hyperactive-impulsive type. Subjects were unmedicated, or they had refrained from taking methylphenidate for 24 hours before testing. Subjects taking other psychotropics were not included in the study. Also, subjects which had suffered a head injury with subsequent loss of consciousness, subjects suffering from neurological or systemic medical diseases, and subjects having symptoms of psychosis were excluded from the study. 42 ADHD subjects graduated from elementary or vocational school, 27 from a secondary school and 5 from university. 1 subject did not specify his education. 67 ADHD subjects were right-handed, 7 ADHD subjects were left-handed, and one ADHD subject was ambidextrous. The ADHD sample partly overlaps with the ADHD sample of a previous study by the authors [29]. In contrast to this previous study, the above ADHD subjects all met the full threshold DSM-IV criteria for ADHD at the time of testing.

The control group consisted of 75 age- and sex-matched healthy subjects recruited from the local community. The mean age in the control group was 36.08 years (SD 8.60). Subjects were recruited by advertising the study in local media, companies and associations. Subjects who had suffered a head injury with subsequent loss of consciousness and subjects suffering from neurological or systemic medical diseases were excluded from the study. Furthermore, control subjects had to score lower than the level of clinical significance on a symptom checklist (Brief Symptom Inventory; [31]). No control subjects were receiving medication at time of testing. 40 control subjects graduated from elementary or vocational school, 24 from a secondary school and 10 from university. 1 subject did not specify his education. 69 control subjects were right-handed, 3 control subjects were left-handed, and 2 subjects were ambidextrous.

For the purpose of verifying classification performance based on the main samples described above, a new sample was recruited. The ADHD validation sample consisted of 6 male and 11 female subjects, who were tested at the Neuropsychological Unit of the Helgeland Hospital in Mosjøen, Norway. The subjects were recruited from four psychiatric outpatient clinics in Helgeland hospital and from doctors in the same region. The age range was 18 to 47 years, the mean age was 30.5 years (SD 9.4). Inclusion in the group was based on the DSM-IV criteria for ADHD, and exclusion criteria corresponded to the criteria used for the main ADHD group with regard to both medication and existing diseases.

The study was approved by the competent ethics committees in Switzerland (ethics committee Grisons) and Norway (REK nord). Written informed consent was obtained from all participants after the procedure had been explained to them.

### Questionnaires and clinical interview

ADHD symptoms were assessed by means of Barkley's Current Symptoms Scale and Childhood Symptoms Scale for retrospective recall of childhood symptoms [30]. These scales were self-administered and completed by the subjects (self report forms) as well as by their partners and parents, if available (other report forms). Each of the scales contains the 18 ADHD symptom items from DSM-IV. The odd-numbered items assess the frequency of inattentive symptoms and the even-numbered address hyperactive/impulsive symptoms on a scale ranging from 0 (never or rarely) to 3 (very often). These two lists of items were scored separately by counting the number of items that have been answered 2 (often) or 3 (very often). The Childhood Symptoms Scale has not been used in the control and ADHD validation groups.

In order to measure current psychological distress and symptoms in both the patient and non-patient samples, the Brief Symptom Inventory [31] was applied. The BSI is a short form of the Symptom Checklist 90-R [32]. The 53-item self-report scale is used to measure nine primary symptom dimensions (somatization, obsessive-compulsive, interpersonal sensitivity, depression, anxiety, hostility, phobic anxiety, paranoid ideation, psychoticism) and three global indices (Global Severity Index, Positive Symptom Distress Index, and Positive Symptom Total). The BSI measures the patient's experience of symptoms in the past seven days. Answers are on a 5-point scale, from 0 (not at all) to 4 (extremely). The Global Severity Index (GSI) measures the overall psychological distress level and is calculated by dividing the cumulative value by the number of answered items. The Positive Symptom Total (PST) defines the number of self-reported symptoms (answer > 0) and the Positive Symptom Distress Index (PSDI) quantifies the symptoms' intensity (cumulative value/PST). Cutoff values used for excluding control subjects were determined by comparison to age-appropriate norms. GSI T scores of 63 or above are considered clinical, as are cases in which two of the dimension scores reach 63 or above.

The subjects' health history was assessed using the Health History questionnaire compiled by Barkley and Murphy [30]. The form lists different types of health problems, and subjects are asked to specify whether they have ever experienced any of these problems. Further, the form asks about the current intake of medication.

Criteria for inclusion in the ADHD group were assessed during a structured clinical interview for adults with ADHD [30]. The interview comprises of an assessment of current and childhood DSM-IV ADHD symptoms, the history of problems at school, the psychiatric history (including drug and medication use), as well as past and present comorbidities.

### Behavioral task

The task is a modification of the visual two-stimulus go/no-go paradigm and has been used for examining the electrophysiological mechanisms of executive operations on various occasions [9,23]. Three categories of visual stimuli (pictures of animals, plants and humans) were presented in 400 trials each consisting of the presentation of a pair of stimuli: animal-animal (go trials), animal-plant (nogo trials), plant-plant (ignore trials), and plant-human (novel trials). In the novel trials, the pictures of humans were presented together with an artificial sound. A detailed description of the presentation modalities can be found in a previous article of the authors [29]. The task was to press a button as fast as possible in response to all go trials.

In trials with a picture of an animal presented as the first stimulus the subject is supposed to prepare to respond. This preparatory set is referred to later as "continue set". In trials with a picture of a plant presented as the first stimulus the subject does not need to prepare to respond. This preparatory set is referred to as "discontinue set".

The responses of the subjects were recorded on a separate channel on the amplifier. The averages across trials for response latency and for response variance were calculated individually for each subject. The number of omission errors (failure to respond in go trials) and of commission errors (failure to suppress a response in nogo trials) were also individually computed for each subject.

### Procedure

The procedure has been described in detail elsewhere [29]. Questionnaires were completed by the ADHD subjects prior to the first session. Then, the ADHD subjects were tested in a first session which comprised a comprehensive, structured clinical interview [30]. The interviews were conducted by trained psychologists. Subsequently, EEG data was recorded in eyes-closed and eyes-opened resting conditions as well as while subjects were performing a visual continuous performance task, which is the focus of this paper. Additional neuropsychological tasks, administered in a second session, are not relevant to this paper.

Control subjects were tested in a single session. After filling out a series of questionnaires, EEG data was acquired. Lastly, the subjects were given a working memory task, whose resulting data is not relevant to this paper.

EEG was recorded using a Mitsar 201 (Mitsar Ltd.) and sampled at 250 Hz with a bandwith of 0.5 to 50 Hz. Impedance was kept below 5 KOhm for all electrodes. Electrodes were placed in accordance with the International 10-20 system using an electrode cap with tin electrodes (Electro-cap International Inc.). Linked ears reference montage was changed to average reference montage prior to data processing. Artefacting was accomplished according to the procedure described in our previous paper [29].

### Independent ERP components

The goal of Independent Component Analysis (ICA) is to utilize the differences in scalp distribution between different generators of ERP activity to separate the corresponding activation time courses [21]. ICA decomposes multichannel ERP signals into a sum of temporally independent and spatially fixed components. Components are constructed by optimizing the mutual independence of all activation time curves, leading to a natural and intuitive definition of an ERP component as a stable potential distribution which cannot be further decomposed into independently activated sources.

The assumptions that underlie the application of ICA to individual ERPs are as follows: 1) summation of the electric currents induced by separate generators is linear at the scalp electrodes, 2) spatial distribution of the components' generators remains fixed across time, 3) each source signal can be modeled as an independent and identically distributed process [21,33]. In addition, we suggest that cortical locations are similar among individuals, so that it is viable to implement the ICA on an array of ERPs for a group of subjects. The details of the ICA method used for this paper are described elsewhere [29]. Grand average ERPs as well as independent components (ICs) were constructed in response to the second stimuli of the trials, in a 1 second time interval after the presentation of the second stimuli. The ICA of the individual ERPs was made separately for each continue (go and nogo conditions) and discontinue (novel and ignore conditions) sets on the basis of the total sample data.

As in any iteration procedure, different datasets (such as a healthy control group and an ADHD group) do not necessarily provide the same ICs. To assure that the components separated in the present study are stable and, consequently, reflect common features of ERPs, we first constructed ICs for the control and the ADHD dataset separately. For further analysis, we only chose ICs which were present in both groups. ICs separated in the respective groups were assumed to correspond to each other if the correlation coefficients of both their topographies and activation curves were higher than 0.5.

For decomposing the individual ERPs of the validation sample into independent components, the same spatial filters which had been constructed for the main sample were used.

### Locating the generators of the independent components

The sLORETA [20] imaging approach was used for locating the generators of the independent ERP components extracted in this study.

### Classification

Classification of subjects into ADHD and control subjects is based on features derived from the individual IC activation curves, comprising latency as well as maximum/minimum and average amplitude values. The automated feature extraction procedure used in this study corresponds to the procedure used in our previous work [29].

In order to reduce the number of possible features, for each of the ICs only the time courses at the site showing the most distinct grand average amplitudes were entered in the feature selection procedure. As to continue- and discontinue-set-specific ICs, only the activation curves of the dominating condition were entered in the feature selection procedure. Go/nogo (continue-set) components 4 and 8 (see results section) showed similar amplitude values in both conditions and therefore the activation curves of both conditions were selected for feature extraction. As to the preparatory-set-independent ICs, activation curves of the ignore condition were selected for feature extraction.

Classification was performed by means of a support vector machine (SVM). Support vector machines are classifiers which originate in machine learning. The supervised learning algorithm seeks to provide the best possible separation of predefined groups in a multidimensional space on the basis of a number of training samples. A specific description of the support vector machine used in this study can be found elsewhere [29]. More extensive information about SVM is provided by Hastie et al. [34].

The presented support vector machine builds a classifier based on a given set of features. However, being based on different feature types and ICs, the number of extracted features for each subject is very large and would result in an overfitting of the SVM classifier. To select an appropriate set of features the predictive power of the resulting classification routine has to be considered. A popular technique to assess how the results of a statistical analysis will generalize to an independent data set is cross-validation [35]. Data is split k-times for an estimation of the performance of each classifier: k-1 parts of the data are used for training each classifier and the remaining part is used to test the predictive power. In this work, a 10-fold cross-validation was implemented.

Validation of the predictive power of the classifier was an important goal of this study. Accordingly, the classification accuracy was further validated by training the SVM on the basis of the main samples data and testing the classifier on the basis of the independent ADHD validation sample data.

### Statistical analysis of behavioral and questionnaire data

The Student's t-test was used in order to assess statistical significance of the group differences related to reaction time and reaction time variance in the behavioral task. Because the variables were not normally distributed, the Mann-Whitney U test was used for assessing statistical significance of the differences related to the number of omission and commission errors in the behavioral task and related to the questionnaire data.

## Results

### Clinical scales

Mean and p-values related to the clinical scales are presented in table 1. Compared to the control group, the ADHD group reported a presence of more current inattention symptoms (*Z *= -11.2, *p *<.001), more current hyperactivity/impulsivity symptoms (*Z *= -10.8, *p *<.001), and more current total ADHD symptoms (*Z *= -11.0, *p*<.001). Correspondingly, the ADHD validation group reported a presence of more current inattention symptoms (*Z *= -8.4, *p *<.001), more current hyperactivity/impulsivity symptoms (*Z *= -8.1, *p *<.001), and more current total ADHD symptoms (*Z *= -7.8, *p *<.001) compared to the control group. The main ADHD group and the ADHD validation group did not significantly differ regarding the number of symptoms on the Current Symptoms Scale.

**Table 1:** Mean (sd) values of the clinical scales

	Controls		ADHD		ADHD validation	
Age	36.08	*(8.60)*	36.05	*(8.42)*	30.53	*(9.39)*
Gender (m/f)	38/37		38/37		6/11	
Handedness (r/l/a)	70/3/2		67/7/1		16/1	
Current inattentive symptoms	***0.09	*(0.34)*	6.53	*(1.70)*	6.12	*(2.91)*
Current hyperactive/impulsive symptoms	***0.09	*(0.29)*	4.92	*(2.32)*	5.12	*(3.08)*
Current ADHD symptoms	***0.18	*(0.49)*	11.45	*(3.06)*	11.24	*(5.53)*
BSI somatization mean	***0.16	*(0.21)*	0.77	*(0.70)*	*1.16	*(0.71)*
BSI obsessive-compulsive mean	***0.32	*(0.29)*	2.15	*(0.84)*	1.90	*(0.85)*
BSI interpersonal sensitivity mean	***0.25	*(0.27)*	1.88	*(1.00)*	***0.93	*(0.67)*
BSI depression mean	***0.10	*(0.19)*	1.42	*(0.98)*	1.24	*(0.80)*
BSI anxiety mean	***0.20	*(0.20)*	1.45	*(0.70)*	*1.12	*(0.74)*
BSI hostility mean	***0.21	*(0.19)*	1.40	*(0.81)*	1.14	*(0.94)*
BSI phobic anxiety mean	***0.04	*(0.09)*	0.70	*(0.69)*	0.80	*(0.77)*
BSI paranoid ideation mean	***0.13	*(0.21)*	1.38	*(0.88)*	*0.92	*(0.88)*
BSI psychoticism mean	***0.06	*(0.12)*	1.23	*(0.79)*	***0.55	*(0.70)*
BSI Global Severity Index	***0.17	*(0.13)*	1.38	*(0.63)*	1.13	*(0.62)*
BSI Positive Symptom Distress Index	***0.96	*(0.33)*	2.18	*(0.50)*	2.03	*(0.43)*
BSI Positive Symptom Total	***8.07	*(5.89)*	31.60	*(9.68)*	28.47	*(10.79)*

Standard deviations are shown in brackets. Asterisks indicate significant differences when compared to the ADHD group (*p < .05, **p < .01, ***p < .001).

As for the BSI symptom dimensions and general indices, compared to the control group, the ADHD group showed significantly higher scale and overall values with regard to any measure (p-values to be seen in table 1). Correspondingly, compared to the control group, the ADHD validation group exhibited significantly higher values regarding all BSI measures (not reported). Compared to the ADHD validation group, the ADHD main group showed significantly lower somatization (*Z *= -2.331, *p *<.05), higher interpersonal sensitivity (*Z *= -3.7, *p *<.001), higher anxiety (*Z *= -2.0, *p *<.05), higher paranoid ideation (*Z *= -2.1, *p *<.05), and higher psychoticism (*Z *= -3.5, *p *<.001) scale values.

As to the additional concerns collected in the clinical interview, in the ADHD main group, significant changes to sleep pattern (28.0%), prolonged periods of sadness/depression (25.3%), significant appetite changes (17.3%), excessive fears/phobias (13.3%), and excessive anxiety (12.0%) were reported most frequently. In the ADHD validation group, the distribution of the most frequent concerns was as follows: periods of sadness/depression (58.8%), significant changes to sleep pattern (47.1%), conduct disorder (41.2%), and excessive anxiety (17.6%).

### Behavioral task performance

Table 2 shows the behavioral performance of the participants in the VCPT. The ADHD group showed a significantly higher number of omission errors (*Z *= -5.0, *p*<.001) and commission errors (*Z *= -2.423, *p*<.05), as well as significantly higher standard errors of reaction time mean (*t*(148) = 5.2, *p*<.001), compared to the control group. The groups did not significantly differ in terms of reaction time.

**Table 2:** Mean (sd) values of the behavioral parameters in the VCPT

	Controls		ADHD		ADHD validation	
Number of omission errors	1.20	*(1.74)*	4.56***	*(5.14)*	7.31***	*(7.14)*
Reaction time (ms)	418.48	*(88.95)*	424.17	*(91.79)*	419.50	*(74.29)*
Standard error of RT mean (ms)	8.08	*(2.51)*	11.00***	*(4.19)*	12.23***	*(3.91)*
Number of commission errors	.37	*(.79)*	.72*	*(1.06)*	.47	*(.80)*

Standard deviations are shown in brackets. Asterisks indicate significant differences when compared to the control group (*p < .05, **p < .01, ***p < .001). No significant differences between the ADHD groups.

The ADHD validation group had a significantly higher number of omission errors (*Z *= -3.7 *p*<.001) and a significantly higher standard error of reaction time mean (*t*(89) = 5.4, *p*<.001) in comparison to the control group. The groups did not significantly differ in terms of reaction time and number of commission errors.

The main ADHD group and the ADHD validation group did not significantly differ with regard to any behavioral performance variables.

### Grand average ERPs

**Figure 1:**
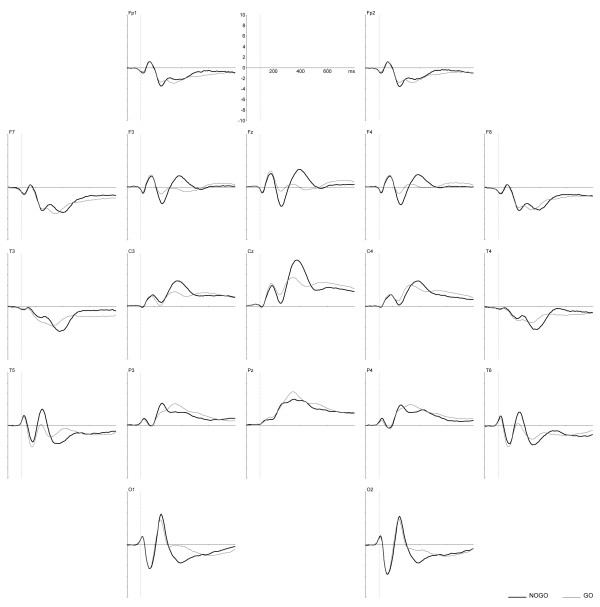
**Go and nogo condition grand average ERPs**. Total group ERPs, assessed in response to the second stimuli of nogo (thick line) and go (thin line) trials. × axis is time in ms, y axis is amplitude in μV.

**Figure 2:**
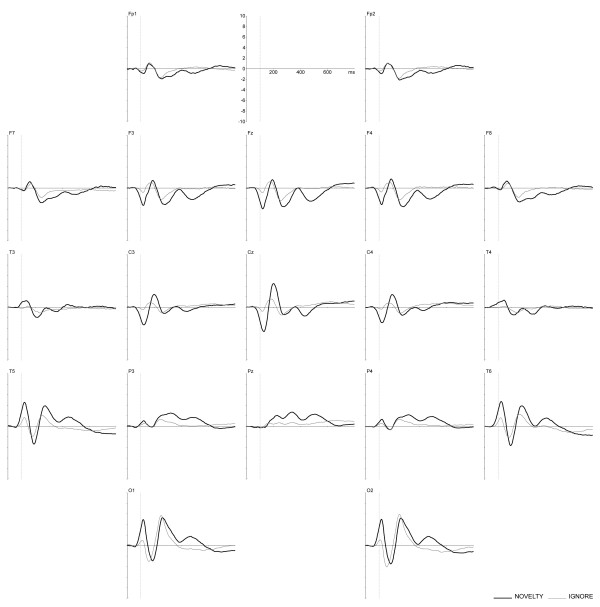
**Novelty and ignore condition grand average ERPs**. Total group ERPs, assessed in response to the second stimuli of novelty (thick line) and ignore (thin line) trials. × axis is time in ms, y axis is amplitude in μV.

The total group grand average ERPs in response to the second stimuli of go and nogo, as well as of novel and ignore condition trials are presented in Figures 1 and 2. The maximum amplitudes of the positive waveforms are found at Pz for go condition with the peak at 345 ms, at Cz for nogo condition with the peak at 370 ms. For novel and ignore conditions, the maximum positive amplitudes are found at the occipital sites at 260 ms and 250 ms respectively.

### Independent ERP components

**Figure 3:**
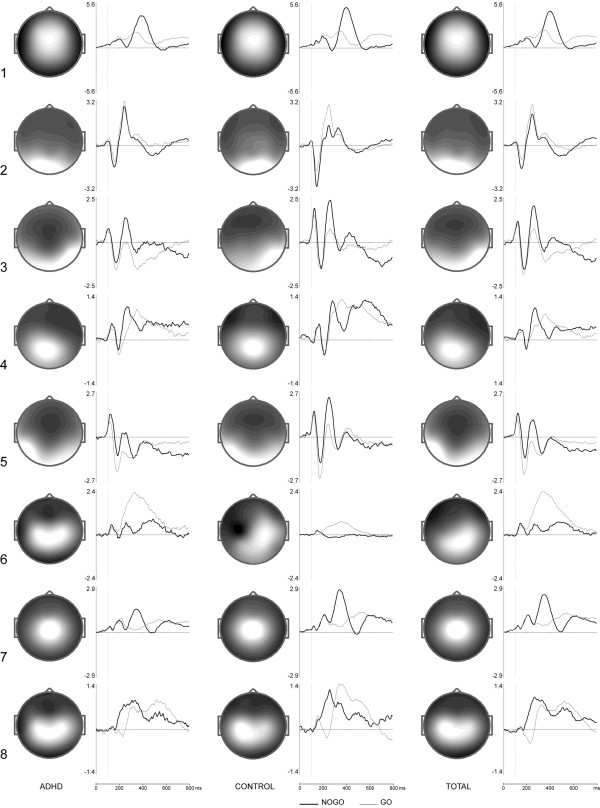
**Topographies and activation curves of go/nogo condition independent components**. ICA was performed on ERPs of the ADHD group (left), on ERPs of the control group (middle) and on ERPs of the total group (right), for a time interval after the onset of the second stimuli in the go (thin line) and nogo (thick line) conditions. × axis is time in ms, y axis is amplitude in standard units.

**Figure 4:**
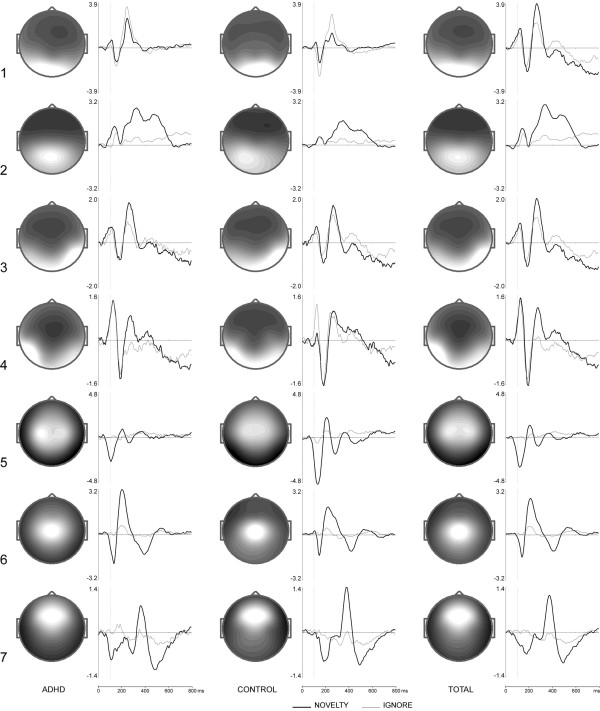
**Topographies and activation curves of novelty/ignore condition independent components**. ICA was performed on ERPs of the ADHD group (left), on ERPs of the control group (middle) and on ERPs of the total group (right), for a time interval after the onset of the second stimuli in the ignore (thin line) and novelty (thick line) conditions. × axis is time in ms, y axis is amplitude in standard units.

Independent components separated by ICA that corresponded to eye movements were excluded from further analysis. Among the remaining components, only those which could be found in both the ADHD and the control group were analyzed. Topographies and activation time courses of the resultant ICs are presented in Figure 3 for go/nogo ICs and in Figure 4 for novel/ignore ICs. As a measure of similarity of the components present in both groups, correlation coefficients obtained for both the topographies and the activation curves were computed (see table 3). The correlation coefficients for the activation curves were computed separately for go and nogo conditions, and for novel and ignore conditions respectively. The labeling of the components ensued according to the localization and the task condition they were extracted from.

**Table 3:** Similarity of the independent components constructed in the ADHD and control groups

Go/nogo independent components				Novelty/ignore independent components			
**IC #**	**Topography**	**Time course**		**IC #**	**Topography**	**Time course**	

		Go	Nogo			Ignore	Novelty
1	0.99	0.92	0.96	1	0.96	0.96	0.88
2	0.90	0.94	0.81	2	0.97	0.63	0.95
3	0.91	0.69	0.84	3	0.99	0.91	0.98
4	0.89	0.86	0.54	4	0.91	0.67	0.36
5	0.89	0.68	0.73	5	0.90	0.50	0.75
6	0.86	0.93	0.05	6	0.75	0.84	0.77
7	0.96	0.85	0.97	7	0.93	0.70	0.80
8	0.78	0.84	0.85				

Correlation coefficients between the independent components of the two groups, computed separately for topography and time course.

**Figure 5:**
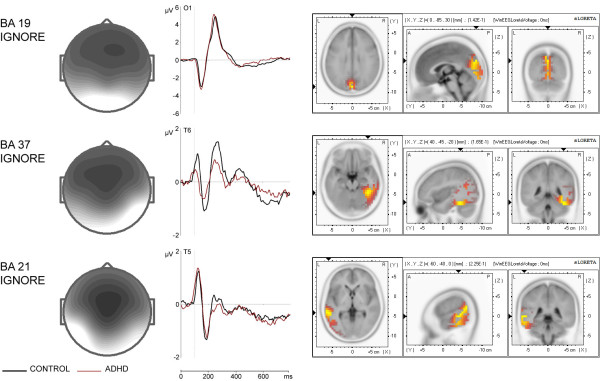
**Topographies and time courses of the preparatory-set-independent components**. Time courses are based on spatial filtration and are depicted separately for control (black line) and ADHD (red line) group. × axis is time in ms, y axis is amplitude in μV. sLORETA imaging of total group is presented on the right.

Figures 3 and 4 show that some of the ICs computed for continue and discontinue sets are found in both preparatory sets. These set-independent components are presented in Figure 5. According to sLORETA, they are distributed over the cuneus of the occipital lobe (BA 19 ignore), over the fusiform gyrus of the right temporal lobe (BA 37 ignore), and over the left middle temporal gyrus (BA 21 ignore). BA 19 ignore component displays a negative peak at 150 ms and a positive peak at 250 ms. The negative deflection resembles the visual N1 wave described in previous ERP studies [36,37]. BA 37 and BA 21 components consist of a sequence of a positive peak at 120 ms, a negative peak at 170 and 180 ms respectively, and a positive peak at about 260 and 270 ms respectively. The prominent negative deflection resembles the occipito-temporally distributed N170 waves described in studies on ERP correlates of object processing [38-40].

**Figure 6:**
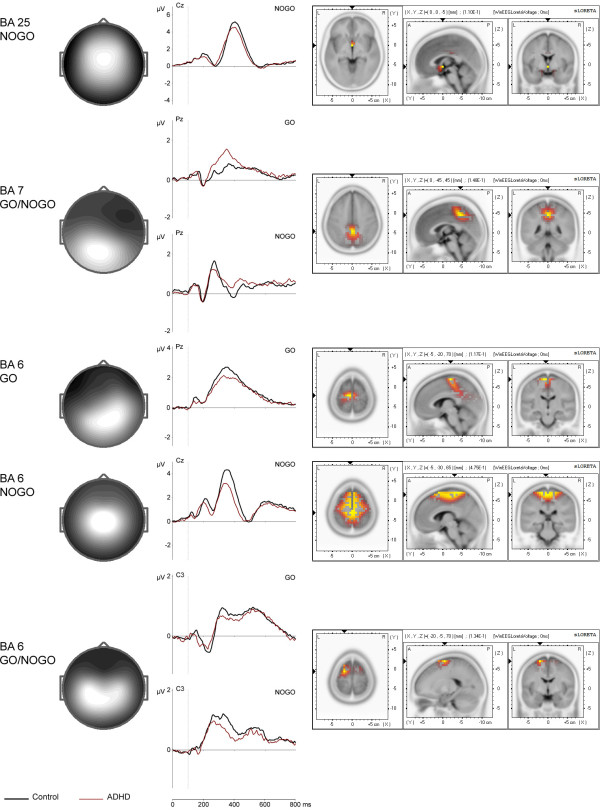
**Topographies and time courses of continue-set-specific (go and/or nogo) components**. Time courses are based on spatial filtration and are depicted separately for control (black line) and ADHD (red line) group. Low-amplitude IC time courses in the non-dominant conditions are not presented. × axis is time in ms, y axis is amplitude in μV. sLORETA imaging of total group is presented on the right.

The continue-set-specific go/nogo components are presented in Figure 6. According to sLORETA, they are located in the anterior cingulate cortex (BA 25 nogo), in the precuneus (BA 7 go/nogo), in the medial frontal gyrus (BA 6 go), in the supplementary motor area (BA 6 nogo), and in the premotor cortex (BA 6 go/nogo). The frontally-centrally distributed BA 25 nogo component shows a negative peak at 270 ms and a positive peak at 400 ms, the negative deflection resembling the conventional N2 nogo wave [41,42]. The parietally distributed BA 7 go/nogo and BA 6 go components both show a positive peak at about 350 ms and match the corresponding parameters of conventional P3b waves, which are elicited in response to target stimuli [43]. BA 6 nogo component is distributed centrally and characterized by three positive peaks, featuring a prominent positive peak at 350 ms. BA 6 go/nogo component is distributed centrally as well and displays a positive fluctuation at 320 ms (go time-course) and 260 ms (nogo time-course) respectively. When comparing go and nogo time-courses, the difference waves of both the BA 6 nogo and BA 6 go/nogo components show characteristics of the conventional nogo-P3 and nogo-N2 waves described by Falkenstein et al. [44].

**Figure 7:**
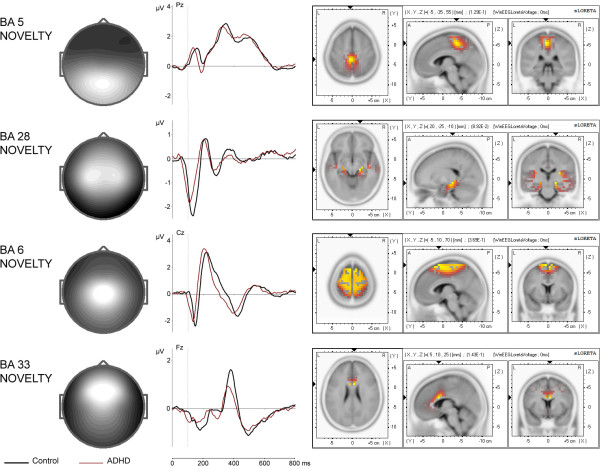
**Topographies and time courses of discontinue-set-specific (novelty) components**. Time courses are based on spatial filtration and are depicted separately for control (black line) and ADHD (red line) group. Low-amplitude IC time courses in the non-dominant ignore condition are not presented. × axis is time in ms, y axis is amplitude in μV. sLORETA imaging of total group is presented on the right.

The discontinue-set-specific ignore/novel components are presented in Figure 7. According to sLORETA, they are located in the supplementary motor area (BA 5 novelty), in the parahippocampal gyrus (BA 28 novelty), in the premotor cortex (BA 6 novelty), and in the anterior cingulate cortex (BA 33 novelty).

BA 5 novelty component shows a prominent positive peak at 330 ms. This deflection resembles the late phase of the P3a ERP component, which is characterized by a peak at 300 to 350 ms after stimulus onset [45]. BA 28 novelty component is characterized by a negative peak at 120 ms and two positive peaks at 200 ms and 370 ms, with the negative deflection resembling the conventional auditory N1 component [37]. BA 6 novelty component shows a negative peak at 140 ms as well as a positive peak at 210 ms, which corresponds to the early phase of the conventional P3a component [45]. Finally, the frontally distributed BA 33 novelty component is characterized by a negative peak at 170 ms and a prominent positive peak at 370 ms.

### Feature extraction and selection

The final feature set was automatically generated using the following feature template settings: The starting point of the IC's time course was 0 ms, the end point was set at 700 ms. Time step size was set at 8 ms, and time window size was 114 ms (+/-25%, and +/-50%). 114 ms corresponds to the median of the deflection's time ranges which were defined as +/- 1.5 standard units from the mean latencies.

The final number of features used for classification was determined by checking the classification performance which was obtained by combinations of up to 10 features. Cross-validation accuracy scores indicate that the predictive power increases up to the fifth feature. The addition of further features does not substantially improve the classifier's performance and increases the risk of overfitting. Therefore, the final feature set was composed of a combination of five features with best classification performance, selected by the automated feature selection algorithm, and it consisted of the following features: minimum peak latency in the 112 to 168 ms range of BA 6 novelty component, minimum peak latency in the 360 to 416 ms range of BA 6 go component, minimum peak amplitude in the 0 to 56 ms range of BA 6 go component, minimum peak latency in the 440 to 496 ms range of BA 6 nogo component, and minimum peak latency in the 192 to 332 ms range of BA 37 ignore component.

### Classification

Cross-validation classification accuracy, using the main samples, was 91% correct classifications (91% sensitivity, 91% specificity). When testing the predictive power by means of the independent ADHD validation sample, classification accuracy was 94%.

## Discussion

In this study, we investigated whether features of averaged ERPs, which were decomposed into independent components by means of ICA, can be used for an accurate classification of ADHD and control subjects. For that purpose, a modification of the visual two-stimulus go/no-go task was used in order to obtain ERP responses from an ADHD and a control sample in four task conditions. The individual ERPs of the ADHD and the control sample were separately decomposed into independent components. Independent component analysis was applied to go/nogo condition ERPs on the one hand, and to novelty/ignore condition ERPs on the other hand. In the respective samples, twelve highly correlating independent components were found, with three of them being task-condition-independent, five being go/nogo-condition-specific, and four being novelty-condition-specific. These independent components were then reconstructed on the basis of the total sample data and provided a set of latency and amplitude features, which were used in the classification procedure. Classification of the adult participants into ADHD and control subjects was performed using a support vector machine. Using a 10-fold cross-validation procedure, classification accuracy was 91%. This accuracy in the discrimination between ADHD and control subjects is remarkable considering the wide age range of the subjects participating in this study and bearing in mind that various authors reported an apparent decline in the diagnostic utility of electrophysiological measures with increasing subject age [24,46,47].

This result is consistent with the classification accuracy obtained in an earlier study [29]. In that earlier study, independent component topographies originating from a big set of healthy subjects have been used as generic spatial filters for decomposing individual ERPs into independent components. In the present study, independent components were genuinely constructed on the basis of the ADHD and control sample ERPs. In doing so, a possible spatial variability between ADHD and healthy subjects was taken into consideration. ICA in the ADHD and control groups resulted in a number of highly correlated independent components, which points towards a high reliability of the independent component's separation.

To our knowledge, there are no other studies with the aim to classify adult ADHD patients and control adults on the basis of ERP data. There are a few studies on children, whose classification accuracies are considerably lower [24,47-49]. In contrast, the result of the present study indicates that the classification of ADHD patients on the basis of ERPs is feasible. However, the separation of ADHD patients from healthy counterparts might be relatively easy when compared to the differential diagnostic categorization of patients. Therefore, further studies gathering ERP data from different patient groups are needed in order to assess differential diagnostic classification accuracies. Another issue which deserves attention concerns the composition of the ADHD samples. Due to comparatively long wash-out periods associated with certain psychotropics, such as SSRI, ADHD patients who take psychoactive medication different from methylphenidates were not included in the study. This group of patients constitutes a considerable part of the adult ADHD population, thus the generalizability of the findings may be limited. Furthermore, potentially confounding variables such as socio-economic standing or intelligence have only marginally been taken into consideration.

A second aim of the study was to test the validity of the classifier. To do so, the SVM was trained with the main sample and the predictive power of the classifier was tested using an independent ADHD sample, which resulted in a classification accuracy of 94%. This result shows that the classifier, based on the ERP feature set identified in this study, is not specific to the study sample and can be applied to any ADHD patients examined in different places. However, the absence of an independent healthy validation sample may constitute a limitation to the validation procedure. Along with a consequential lack of specificity estimation, anomalies in the control group could have an effect on classification accuracy.

In spite of the limitations mentioned above, the findings of the present study indicate that event-related potentials can make a significant contribution to the diagnosis of ADHD. However, in terms of personalized medicine, associating independent ERP components with a functional meaning can increase their utility as biomarkers by allowing well-directed clinical interventions. Three types of independent ERP components were identified in the present study: task-condition-independent components, go- and nogo-condition-specific components, as well as novelty-condition-specific components.

The task-condition-independent components were localized in occipital and occipito-temporal areas. In view of these sources and given that all task conditions involve the presentation of visual stimuli, the condition-independent components are assumed to reflect stages of the visual information flow.

The go and nogo conditions (continue set) require the exertion of executive operations such as the monitoring and inhibition of actions, which have mainly been related to the frontal lobe [50]. The continue-set-specific (CS) components could, except for BA 7 go/nogo component, in fact be localized in frontal brain areas. The BA 25 nogo component was localized in the anterior cingulate cortex and shows a negative peak at 270 ms. This negative peak may be related to the N2 nogo wave [41,42], a wave emerging when ERPs elicited by nogo trials are compared with ERPs elicited by go trials. The N2 nogo wave has been associated with response inhibition [51] and conflict monitoring [52]. The parietally distributed BA 7 go/nogo and BA 6 go components both feature characteristics of conventional P3b waves, which, according to the dominant view, are associated with context-updating and memory operations [43]. Other authors link the P3b component to a monitoring process that mediates between perceptual analysis and response initiation [53] or to mechanisms involved in event categorization [54]. BA 6 nogo as well as BA 6 go/nogo component show characteristics of the conventional N2 nogo and P3 nogo waves, which both have been associated with the mechanism of inhibition [44]. The association with the process of inhibition is supported by the localization of these components in the left premotor cortex (BA 6 go/nogo) and in the supplementary motor area (BA 6 nogo), a part of the cortex which has been demonstrated to be involved in motor inhibition [55].

As for the discontinue set (i.e. novel and ignore conditions), four independent components could be identified which were present in both the ADHD and the control group. They all were dominant in the novelty condition. In the novelty condition, a novel sound is presented along with the second visual stimulus. Novel auditory (or visual) stimuli, which are not of relevance to the performance of the task, elicit a characteristic ERP component named P3a or novelty P3. The P3a component has been linked to different processes such as the orienting response or the detection and evaluation of novelty [45,56,57]. Two models have been proposed to explain the functional significance of the P3a component. According to the attention-switching model, the P3a component reflects the involuntary switch of attention to deviant stimuli, which distract the person [58]. According to the response inhibition model, on the other hand, the novelty P3 component reflects the inhibition of a response engaged automatically with the detection of a deviant event [59]. As for the independent components identified in the present study, BA 5 novelty component was localized in the paracentral lobule, posterior to the primary somatosensory areas. Its features are similar to the late phase of the P3a ERP component, which is thought to reflect orienting of attention towards novelty [60]. BA 6 novelty component is characterized by a prominent positive peak at 210 ms, which corresponds to the early phase of the conventional P3a component reported in various studies [60,61]. This early P3a subcomponent has been assumed to reflect the breaking of regularity in the environment [60]. However, localized in the premotor cortical area, BA 6 novelty component may, according to the response inhibition model of Goldstein et al. [59], reflect the inhibition of a deviance response, which is triggered by such violation of the environmental model. The BA 33 novelty component was localized in the anterior cingulate cortex. In a study by Dien et al. [62], the anterior cingulate cortex has been shown to correspond to the source of the novelty P3. Further, Goldstein et al. [59] report an independent fronto-central P3 novelty component which shows characteristics that are very similar to BA 33 novelty component. Therefore, this component seems to be associated with the detection of novelty as well. The BA 28 novelty component was localized in the parahippocampal gyrus and characterized by a prominent negative peak at 120 ms and two positive peaks at 200 ms and 370 ms. The negative deflection corresponds to the conventional auditory N1 component which has been found in numerous studies [37]. The association of this component with the processing of auditory input is confirmed by findings from Boutros et al. [63] who, using implanted electrodes, found a negativity at around 100 ms in the posterior hippocampus as well as a positivity at around 400 ms in the rhinal cortex in response to auditory stimuli.

The feature set which was used for classification and which discriminated best between ADHD and control subjects primarily consisted of features derived from independent components presumably associated with the inhibition of an action (BA 6 novelty, BA 6 nogo), on the one hand, and with the parietally distributed P3b wave (BA 6 go), on the other hand. However, these features which in combination allow for a discrimination of the ADHD and control groups do not necessarily produce significant group differences when considered as separate features. Nevertheless, abnormalities in action inhibition correspond to Barkley's [7] influential model of ADHD. According to this model, the disorder is characterized by a deficit in behavioral inhibition, which is supposed to be a superordinate executive function setting the occasion for the occurrence of other executive functions. According to a cognitive model proposed by Polich [43], inhibition of on-going activity can facilitate transmission of stimulus information from frontal to temporal-parietal areas related to P3b production to promote memory operations. Consequently, deficits in inhibitory processes could result in constraints regarding other executive functions and subsequent memory processing.

## Conclusions

This study shows that event-related potentials can substantially contribute to the diagnosis of ADHD in adults. Furthermore, ERP biomarkers can objectify and personalize the disorder. The use of independent ERP components facilitates a precise determination of abnormalities with regard to cortical localization and temporal occurrence, and consequently provides an indication of their functional meaning. The method can successfully be used in clinical practice and enables the implementation and further development of personalized medicine.

## Competing interests

GC, VAG, VAP, GMB report no potential conflicts of interest.

AM and JDK are members of the board of HBImed company. HBImed provides accesses to a referential database.

## Authors' contributions

AM, GC, JDK, VAG contributed to the study conception and design. GC, VAG, AM headed the acquisition of data. GC, AM, JDK, GMB, VAG, VAP have been involved in drafting or revising the manuscript. GMB and VAP made substantial contributions to the analysis of data by implementing support vector machine (GMB) and ERP decomposition by means of ICA (VAP). All authors read and approved of the final manuscript.
